# Electronic surveillance of outbreaks in Lebanon

**DOI:** 10.1186/1753-6561-2-s3-s2

**Published:** 2008-11-14

**Authors:** Nada Ghosn, Alia Nasredine, Yolla Merhy Baddour, Denis Coulombier, Samah Nasserdine

**Affiliations:** 1 Lebanese Ministry of Public Health, Beirut, Lebanon; 2 World Health Organization/Lyon office

## Abstract

**Background:**

This paper describes and assesses the electronic surveillance of outbreaks based on the early warning for four endemic diseases – typhoid fever, amebic dysentery, viral hepatitis A and brucellosis – in Lebanon, for the first 28 weeks of 2005 and first 26 weeks of 2007.

**Methods:**

The electronic early warning system is based on the mandatory notification of 37 targeted diseases. The four target diseases assessed in this paper are based on monthly notification. Standards were set for case definitions and forms. Physicians and hospitals report to the Ministry of Public Health (MOPH), where data is checked and transmitted to a central location for entry into the national database, which stores historical and current data, as well as population estimates based on national surveys. The event date was selected for case dating. Indicators triggering abnormalities include number of cases, rates, and relative ratios. Four relative ratios were selected using the period of 1 week, 4 weeks or 52 weeks for the current and previous years. Screening was conducted on a weekly basis in 2005, and on a daily basis in 2007. Abnormal signals were verified, documented and grouped by alert-episodes for each disease, district, and period. MOPH teams verified and investigated case clustering.

**Results:**

During the first 28 weeks of 2005 and the first 26 weeks of 2007, screening operations were 68% and 89%, respectively, for completeness. Detected abnormal signals were 26 and 166 and identified alert-episodes were 11 and 22, respectively. Verified clusters were 7 and 11; positive predictive value for clusters identification was 64% and 50%, respectively. The time interval between first cases and first abnormal signals was on average 4 weeks and 5 weeks, respectively.

**Conclusion:**

Timely reporting, transmission, data entry, analysis and communication are the elements of timely outbreak detection. The electronic surveillance of outbreaks for epidemic-prone diseases, which are mandatory notified on a monthly basis using indicator-based thresholds, is capable of detecting spatio-temporal clusters and outbreaks; however, with some delay. The national surveillance system needs to be reviewed in order to provide timely data for early warning surveillance and response.

## Background

Epidemiologic surveillance for communicable diseases was re-initiated in Lebanon in 1995. Historically, surveillance was initiated by the Law of 31 December 1957, but it was interrupted during the civil war from 1975 to 1994. Electronic surveillance is a necessary tool for storing, analyzing, displaying, mapping, generating abnormal indicator-based signals, modeling, disseminating, reporting and standardizing data/information. This paper will describe and assess [1,2] the electronic surveillance of outbreaks for four endemic diseases – typhoid fever, brucellosis, viral hepatitis A (VHA), and amebic dysentery [3] – in Lebanon for the first 28 weeks in 2005 and the first 26 weeks in 2007.

## Methods

### Purpose

The purpose of an electronic early warning system is the generation of abnormal indicator-based signals indicative of events of public health importance, routine automatic screening at all levels on a weekly/daily basis, and early outbreak detection.

### Stakeholders

Physicians and hospitals are the primary data providers. Since the end of 2001, public and private hospitals have designated the main staff member in charge of disease notification to the Ministry of Public Health (MOPH). First-line data users are MOPH surveillance officers at central and peripheral levels.

### Operations

Operations are based on two systems: mandatory notification and electronic early warning.

#### Mandatory notification

Mandatory notification targets 37 diseases, which are immediately or monthly notifiable. Standard case definitions are reviewed for each disease by MOPH circulars based on WHO recommendations [4-8]. One reporting standardized form is completed by the physician or hospital representative. Standard specific investigation forms are completed by a MOPH surveillance staff member. Physicians can report to the MOPH either at the district, province or central level. Reporting forms are usually sent to the MOPH by facsimile. Since 2007, physicians and hospitals were encouraged to report to MOPH through the mail using postage paid envelopes. Forms are transmitted from peripheral levels to the central level on a monthly basis. Since 2007, forms have to be transmitted on a weekly basis. On average, 1345 cases of typhoid fever, VHA, amebic dysentery and brucellosis were reported annually to the MOPH for the period of 2004–2006. Among them, 95% were notified by hospitals. Upon receipt at the MOPH, forms are marked with the date of receipt. Data completeness and laboratory findings are verified. When necessary, data is completed by contacting data providers by phone. Data entry for case-based surveillance is performed at the central level into the Epidemiological Surveillance Unit (ESU) database, a local application that was developed using the following public software: EpiData 2.1 b, Epi Info 6.04 d and Winglue [9-11]. The database includes coded variables for disease, time, place, and person. According to available dates, a disease-event is dated, by order of priority, by: 1) onset; 2) diagnosis; 3) admission; 4) reporting; 5) reception (Table 1).

**Table 1:** Distribution (%) of cases according to selected date for disease-event for reported cases of typhoid fever, dysentery, viral hepatitis A and brucellosis, in Lebanon, Jan 2005 to Jun 2007.

**Priority Choice Order**	**Dates**	**First Semester 2007**	**Year 2006**	**Year 2005**
1	Date of onset	65.1	70.7	72.5
2	Date of diagnosis	23.6	24.1	20.1
3	Date of hospital admission	2.9	1.9	1.4
4	Date of reporting	8.2	3.1	1.0
5	Date of receiving report	0.3	0.3	4.9

	**Total**	**100**	**100**	**100**

#### Electronic early warning system

The electronic early warning system screens the national database searching for abnormal signals, using the "EWARN" application [12]. Two data sources are used:

• ESU database, which provides the historical data (starting year 1995) and current year data.

• Population estimates based on national surveys of 1994–1996, 1997, 2004, and UN national growth rates [13-16].

Database files are compiled into one aggregated file.

### Time

Cases are computerized according to the week of disease-event. Different mobile time periods are used as a frame for comparison: current week, current 4 weeks, current 52 weeks, 3 weeks previous to current week, 48 weeks previous to current 4 weeks, same 4 weeks of last year, same 52 weeks of last year.

### Indicators and thresholds

Indicators for triggering abnormalities include absolute numbers, relative increase ratios and incidence rates (weekly and 52-week period). Relative ratios are:

• "RR1/3": cases for current week over weekly average of previous 3 weeks.

• "RR4/48": cases for current 4 weeks over 4-week average of previous 48 weeks.

• "RR4/4": cases of current 4 weeks over cases of same 4 weeks one year ago.

• "RR52/52": cases of current 52 weeks over cases of same 52 weeks one year ago.

The threshold for relative increase is set to 1.5 if the denominator is >0. Otherwise, an abnormal signal is generated for at least 3 cases.

### Data outputs

For every area, generated abnormal signals are listed specifying the disease and observed abnormality. Additional outputs include disease-tables, area-tables, epidemic curves, and maps with Epimap2.0 [17]. All outputs are in HTML format allowing easy navigation between pages.

### Epidemiologic analysis, interpretation and investigation

Routine screening was conducted on a weekly basis in 2005, and on a daily basis since the end of 2006. At each new screening, outputs are archived for later review.

### Abnormal indicator-based signals

Abnormal indicator-based signals are identified by disease, place, and time. One abnormal signal may include one or several indicators. The indicator-based characteristic refers to the use of structured data collected through routine surveillance systems as mandatory notification [18].

### Alert-episode

Alert-episode is a group of signals for one disease that reflects common time and place findings. An alert-episode has starting and ending dates. An alert-episode is used to highlight the potential occurrence of clusters and outbreaks.

### Cluster

An alert-episode presenting acute change – related to current week or 4 week indicators – is considered to reflect a potential cluster. Cluster is defined by the following criteria:

• Person: at least three cases or an increasing number

• Time: during current 4 weeks

• And one of the following:

• Place: common/adjacent localities

• Social: common social link (institution, gathering, etc.)

Cluster verification is done by collecting information in-office. The in-office verification includes: checking for duplicates and data, mapping cases at locality level with HealthMapper2.0 [19], completing laboratory findings, and phone-interviewing cases using investigation forms. A verified cluster is flagged for in-field investigation.

### Documentation

In 2005, each abnormal indicator-based signal was archived in "ASTER," a local database. Since 2007, abnormal signals are listed in several sheets:

• Matrix by time and place for monitoring signals and detecting alert-episodes;

• Alerts log book for identifying and following alert-episodes;

• Daily sheet for descriptive analysis by disease, time, place, and person.

### Surveillance staff

The central team is in charge of managing the electronic early warning system, including communicating and assisting peripheral teams regarding potential alerts. The peripheral teams are in charge of verifying alert-episodes and clusters, and conducting investigations.

## Results

### First period

During the first 28 weeks of 2005, 19 weekly screening operations were conducted. Twenty-six weekly abnormal signals were detected. For those signals, 11 alert-episodes were identified: 5 for typhoid fever, 2 for amebic dysentery, 3 for brucellosis and 1 for VHA. Among the alert-episodes, 7 clusters were verified. Three of them were related to outbreaks of typhoid fever because of water contamination (Table 2).

**Table 2:** Alert-episodes identified through electronic early warning in Lebanon for the period from 2005-W01 to 2005-W28.

**#**	**Disease (ICD-10)**^a^	**District**	**Abnormal Signals**			**Cases**				**Cluster**^c^
				
			**Starting Week**	**Ending Week**	**No. of Weeks**^b^	**Cases Count**	**Starting Week**	**Confirmed Cases**	**No. of Affected Localities**	
1	A01	Chouf	W04	W04	1	3	W03	0	2	No
2	A01	Rashaya	W10	W26	8	23	W08	0	11	Yes
3	A23	Baalbeck	W13	W13	1	5	W05	0	3	No
4	A06	Kesrwan	W20	W26	5	10	W16	6	4	Yes
5	A23	Nabatieh	W21	W21	1	4	W17	0	2	No
6	A01	Aley	W22	W23	2	12	W19	3	4	Yes
7	B15	Chouf	W22	W23	2	5	W21	3	3	Yes
8	A01	Menieh-Dennieh	W23	W28	3	49	W17	14	2	Yes
9	A01	Tripoli	W23	W23	1	5	W20	2	1	Yes
10	A06	Beirut	W28	W28	1	3	W23	3	-	No
11	A23	Metn	W28	W28	1	5	W20	0	4	Yes

^a ^International Classification of Diseases – Version 10; ^b ^Number of weeks with abnormal signals; ^c ^Temporo-spatial cluster.

### Second period

During the first 26 weeks of 2007, 133 daily screening operations were conducted over 150 working days and 199 daily abnormal signals were detected. For those signals, 22 alert-episodes were identified: 7 for typhoid fever, 2 for amebic dysentery, 2 for brucellosis and 11 for VHA. Among the alert-episodes, 11 tempo-spatial clusters were verified. One outbreak of typhoid fever and 1 of VHA were found because of water contamination (Table 3).

**Table 3:** Alert-episodes identified through electronic early warning in Lebanon for the Period from 2007-W01 to 2007-W26.

**#**	**Disease (ICD-10)**^a^	**District**	**Abnormal Signals**				**Cases**				**Cluster**^c^
				
			**Starting Week**	**Ending Week**	**No. of Weeks**^b^	**No. of Days**^d^	**Cases Count**	**Starting Week**	**Confirmed Cases**	**No. of Affected Localities**	
1	A06	Baabda	W02	W03	2	7	3	W44	3	3	no
2	A01	Baabda	W02	W03	2	9	9	W38	1	4	yes
3	B15	Tyre	W02	W07	5	22	15	W42	4	10	yes
4	B15	Baalbeck	W05	W07	3	13	6	W02	2	4	no
5	B15	Zahleh	W05	W08	4	17	10	W04	9	5	yes
6	B15	Jezzine	W05	W07	3	13	3	W52	1	1	no
7	B15	Metn	W05	W07	3	13	5	W01	4	6	no
8	A23	Metn	W08	W08	1	1	3	W08	0	3	no
9	A01	Hasbaya	W08	W08	1	1	3	W03	0	2	yes
10	B15	Metn	W09	W14	6	20	8	W08	2	9	yes
11	B15	Baalbeck	W10	W10	1	3	3	W08	1	3	no
12	B15	Baabda	W10	W14	4	9	9	W05	9	6	yes
13	A01	Rashaya	W11	W11	1	1	6	W07	0	3	no
14	B15	Beirut	W11	W11	1	3	3	W07	2	3	no
15	A06	West-Bekaa	W13	W14	2	3	11	W03	11	6	yes
16	A01	Hermel	W15	W18	4	11	5	W11	0	2	yes
17	B15	Baalbeck	W17	W20	4	12	10	W12	5	6	yes
18	A23	Zahleh	W19	W20	2	3	5	W15	0	4	no
19	A01	Batroun	W21	W25	5	23	4	W18	0	4	no
20	A01	Zahleh	W21	W21	1	1	10	W19	0	3	no
21	B15	Metn	W21	W23	3	5	6	W18	4	3	yes
22	A01	Marjeoun	W24	W25	2	9	8	W22	0	3	yes

^a ^International Classification of Diseases – Version 10; ^b ^Number of weeks with abnormal signals; ^c ^Temporo-spatial cluster; ^d ^Number of days with abnormal signals.

### Indicators

The completeness of conducting alerts detection is 68% for the first period of 2005 and 89% for the second period of 2007. The timeliness measured by the time-interval between first cases and first alerts shows an average of 4 weeks for the first period and 5 weeks for the second period. The validity measured by the positive predictive value for the two periods is 64% and 50%, respectively.

## Discussion

### Time definition

The system has a delay in detecting alert-episodes since the first cases occurred. Sources of obstacles for timely detection are:

• Delay in reporting to MOPH since the target diseases are monthly notifiable;

• Delay in transmission from peripheral to central level. Weekly transmission is not respected by all districts;

• Delay in data entry at central level, possibly during holiday periods.

Ways to improve timeliness can be explored by the following:

• Compute cases by week of reception to MOPH, which will reflect timeliness of the "EWARN" application and not of the surveillance system;

• Define epidemic-prone diseases as weekly, even immediately, notifiable;

• Analyze other available timely diseases databases;

• Perform data entry at peripheral level which is linked electronically to the central level;

• Establish electronic reporting at the data-providers level.

### Geographical information system

Cluster definition may not be optimal. The use of GIS can enhance cluster definition and investigation procedures. It helps in visualizing cases and adding new cluster criteria based on exposure: infrastructure (e.g., water, sanitation) and environment (e.g., farms, vectors). Besides ensuring human resources, equipment/material, and budgeting, more efforts are being directed for information resources:

• Ensuring Information and Communication Technology at all levels;

• Training and building capacity of available staff members on how to use information.

According to defined core competencies, the training process is being built upon:

• Issuing documented national guidelines: case definitions, population estimates, maps, ISO-weeks; and descriptive, investigation and monitoring tools;

• Conducting continuous training with exercises;

• Supervising/monitoring.

## Conclusion

The electronic surveillance of outbreaks for epidemic-prone diseases, which are mandatory notified on a monthly basis using indicator-based thresholds, is capable of detecting spatio-temporal clusters and outbreaks; however, with some delay. The rationale of monthly reporting and monthly transmission of data and information is not adapted for early warning engines. The national surveillance system needs to be reviewed in order to provide timely data for early warning surveillance and response systems and epidemic intelligence.

On the other hand, the IHR (2005) requires inclusion of informal sources in surveillance as event-based monitoring. This will detect additional outbreaks and allow measuring the sensitivity of the electronic early warning system.

Surveillance is not static. It is data to be collected, analyzed and displayed in various manners to present relevant intelligible information.

## List of abbreviations used

ESU: Epidemiological Surveillance Unit; EWARN: Early Warning; GIS: Geographical Information System; IHR(2005): International Health Regulations (Revision of 2005); MOPH: Ministry of Public Health; RR: Relative Ratio; UN: United Nations; VHA: Viral Hepatitis A; WHO: World Health Organization.

## Competing interests

The authors declare that they have no competing interests.

## Authors' contributions

Nada Ghosn developed the "ESU" database, established forms, and supervised surveillance team. Alia Nasredine, Yolla Merhy Baddour and Samah Nasserdine conducted the alerts screening, documentation and communication of alerts to peripheral teams.
